# Characterizing unsuccessful animal adoptions: age and breed predict the likelihood of return, reasons for return and post-return outcomes

**DOI:** 10.1038/s41598-021-87649-2

**Published:** 2021-04-13

**Authors:** Lauren Powell, Chelsea Reinhard, Donya Satriale, Margaret Morris, James Serpell, Brittany Watson

**Affiliations:** 1 School of Veterinary Medicine, University of Pennsylvania, Philadelphia, PA USA; 2 Charleston Animal Society, North Charleston, SC USA

**Keywords:** Animal behaviour, Psychology

## Abstract

A considerable number of adopted animals are returned to animal shelters post-adoption which can be stressful for both the animal and the owner. In this retrospective analysis of 23,932 animal records from a US shelter, we identified animal characteristics associated with the likelihood of return, key return reasons, and outcomes post-return for dogs and cats. Binary logistic regression models were used to describe the likelihood of return, return reason and outcome based on intake age, intake type, sex, breed and return frequency. Behavioral issues and incompatibility with existing pets were the most common return reasons. Age and breed group (dogs only) predicted the likelihood of return, return reason and post-adoption return outcome. Adult dogs had the greatest odds of post-adoption return (OR 3.40, 95% CI 2.88–4.01) and post-return euthanasia (OR 3.94, 95% CI 2.04–7.59). Toy and terrier breeds were 65% and 35% less likely to be returned compared with herding breeds. Pit bull-type breeds were more likely to be returned multiple times (*X*_*2*_ = 18.11, *p* = 0.01) and euthanized post-return (OR 2.60, 95% CI 1.47–4.61). Our findings highlight the importance of animal behavior in the retention of newly adopted animals and provide useful direction for allocation of resources and future adoption counselling and post-adoption support services.

## Introduction

An estimated 3.2 million animals are adopted from animal shelters in the US each year^1^, and the rate of adoption appears to be increasing^2^. Most owners report high levels of satisfaction with their newly adopted pet^3–5^, yet a considerable number of adopted animals are returned to shelters for various reasons. Current estimates range between 7 and 20%^6–12^. Over recent years, the perception of returned adoptions within the sheltering community has begun to change^13, 14^. While returns were once viewed as “failed” adoptions, there is now more emphasis placed on the possible benefits of temporary adoptions^15^, such as short-term stress relief^15^ and an increased understanding of the animals’ behavior in the home environment. Nevertheless, the return process can be stressful for both the animal and the owner. The animal’s likelihood of a live release outcome (leaving the shelter alive) may be jeopardized if the shelter does not have the required space or resources available, meaning the animal may be euthanized (humane killing of the animal). Re-entry to the shelter also means the animal is again exposed to the multitude of stressors associated with the shelter environment (see Taylor and Mills^16^ for review). Shore^17^ reported more than half of relinquishing adopters found the return process ‘very difficult’ and 41% indicated they would not adopt another animal in the future^17^.

Behavioral problems are reported as a key reason for unsuccessful dog adoptions worldwide^6, 8, 17, 18^. A recent study of 102 returned dogs at a shelter in Texas found 56% of dogs were returned due to behavioral issues, 31% were returned for owner-related reasons and 9% were returned for medical needs. Aggression towards humans and animals were listed as the two most common return reasons^18^. In the UK, 59% of dogs returned to Dogs Trust shelters occurred due to behavioral problems, with dogs displaying aggression towards people having the highest likelihood of return^8^. Mondelli, et al.^7^ found 39% of returns to a shelter in Italy were attributed to misbehavior, such as barking, destruction and hyperactivity, and a further 15% were returned for aggression. At a shelter in Northern Ireland, Wells and Hepper^9^ found a significantly higher proportion of returned dogs exhibited behavior problems compared with retained dogs^9^.

The primary factors that drive cat returns are less clear cut^5, 18, 19^. Hawes, et al.^18^ found that cats were returned more frequently due to owner-related reasons than animal-based reasons, such as moving, inability to afford basic care and medical needs of the adopter. However, when considering the return reasons individually, aggression towards humans and destructive tendencies were ranked as the second and equal third most common reasons^18^. Other studies have reported behavior as the most prevalent return reason, although allergies to the cat and owner circumstances also led to a number of unsuccessful adoptions^5, 19^. Data regarding postadoption returns of other species are scarce.

The likelihood of post-adoption returns is also associated with a range of owner and animal characteristics. The presence of children in the adopted home has been linked with a higher risk of return^5, 8, 10, 20^. Young owners and first-time owners also comprise a higher proportion of returned adoptions^10, 20^. The risk of return is greater among older animals^5, 19^, male dogs and medium to large dogs^7, 8^. Conversely, caretaking behaviors of owners, such as visiting a veterinarian^20, 21^, allowing the animal to sleep in a family member’s bed and attending training classes, have been associated with a decreased risk of return^8^. Despite the influence of animal and owner variables on the risk of return, there is a lack of data regarding differences in return reasons based on animal or owner characteristics.

There is also a dearth of information about animals’ outcomes post-return. Two previous studies have found that returned dogs had a euthanasia rate between 40 and 50%, although both studies were conducted more than a decade ago^12, 22^. Recent research reported drastically different results with 791 of 816 returned dogs (97%) having a live release outcome. In this study, returned dogs had 4.77 times greater odds of live release compared with owner surrenders. This figure may overestimate the true live release rate of returned dogs as the authors considered adoption, transfer to rescue group, and sent to foster care as a live release outcome. Including the latter as an outcome is inconsistent with their definition of foster care as an interim home for rehabilitation prior to permanent adoption^11^.

Understanding the key factors that result in unsuccessful adoptions across all species will enable animal shelters to develop preventive strategies and target their resources towards the animals and owners who need them most. The aims of this study were to: (a) identify characteristics associated with a greater risk of return; (b) describe the key return reasons and the variations in return reasons by animal characteristics; and (c) examine animals’ outcomes post-return and identify factors that predicted euthanasia.

## Results

Between 2015 and 2019, 23,932 animals were adopted from Charleston Animal Society, including 9996 dogs, 13,450 cats and 486 animals of other species. Of the adopted animals, 9.2% (*n* = 2211) were returned to the animal shelter within six-months of adoption. Dogs were the most frequently returned species with a return rate of 16.3% (*n* = 1628), followed by rabbits at 9.0% (*n* = 15). Cats were returned at a significantly lower rate of 4.2% (*n* = 559, *X*_*2*_ = 997.64, p < 0.001). The remainder of returns consisted of two pigs, four guinea pigs, two mice and one hamster. Table 1 presents the descriptive characteristics of all animals adopted between 2015 and 2019.

**Table 1:** Descriptive characteristics of animals adopted between 2015 and 2019 (*n* = 23 945).

Characteristics	Dogs		Cats	
	Not returned (%, n)	Returned (%, n)	Not returned (%, n)	Returned (%, n)
**Age at intake**				
Puppy/kitten (≤ 6 months)	32.5 (2723)*	14.6 (237)*	76.2 (9817)	48.7 (272)*
Young adult (> 6 months-2 years)	35.9 (3005)	43.8 (713)*	11.5 (1485)	20.6 (115)*
Adult (> 2–8 years)	26.8 (2242)*	37.9 (617)*	10.2 (1320)*	24.7 (138)*
Senior (> 8 years)	4.8 (398)	3.7 (61)	2.1 (269)	6.1 (34)*
**Sex (%, n female)**	48.6 (4064)	46.3 (754)	50.8 (6549)	51.0 (285)
**Length of stay—days (mean, SD)**^**a**^	20.5 (30.7)*	16.6 (17.7)*	35.0 (30.3)*	29.6 (40.2)*
**Breed group**				
Herding	10.0 (825)	9.8 (157)	–	–
Hound	16.1 (1332)	19.8 (328)*	–	–
Non-sporting	1.7 (140)	2.1 (33)	–	–
Pit bull-type	29.6 (2451)	36.4 (586)*	–	–
Sporting	19.0 (1573)	15.7 (252)*	–	–
Terrier	8.1 (669)	5.7 (92)*	–	–
Toy	10.8 (896)*	4.6 (74)*	–	–
Working	4.8 (397)	5.3 (86)	–	–
**Intake type**				
Owner relinquishment	12.3 (1031)	11.1 (181)	9.2 (1187)	14.0 (78)*
Returned adoption	0 (0)	0.9 (14)	0 (0)	0.2 (1)*
Stray	34.6 (2899)	32.5 (525)	57.7 (7440)	49.9 (279)
Seized	45.8 (3835)	48.5 (790)	27.4 (3536)	29.9 (167)
Transfer from another animal shelter	7.2 (603)	7.2 (118)	5.6 (728)	6.1 (34)
**Outcome type**^**b**^				
Adoption	100 (7785)^c^	80.3 (1308)	100 (12,891)^c^	90.2 (504)
Euthanasia	–	14.5 (236)	–	3.2 (18)
Return to owner/guardian	–	2.0 (33)	–	2.3 (13)
Transferred to another animal shelter	–	2.8 (46)	–	2.3 (13)
Died	–	0.2 (3)	–	2.3 (6)
Return to field	–	–	–	0.9 (5)
Other	–	0.1 (2)	–	–

Table shows the breakdown (%) of non-returned or returned animals within each characteristic.

*Indicates there was a statistically significant difference between returned and non-returned animals based on Pearson’s Chi Square test or independent t-tests for length of stay.

^a^Reflects initial length of stay (prior to return) for returned animals.

^b^Reflects the most recent outcome for animals that were returned multiple times.

^c^Only animals that were adopted at least once were included in this study.

Of the animals returned to the shelter, most were returned once (85.7%, *n* = 1895), 11.2% of animals were returned twice (*n* = 220 dogs, *n* = 28 cats) and 2.0% of animals were returned three times (*n* = 44 dogs, *n* = 6 cats). Fourteen dogs were returned four times, three dogs were returned five times and one dog was returned six times. Dogs who were returned once did not differ in sex (*X*_*2*_ = 2.32, *p* = 0.13) or return reason (*X*_*2*_ = 8.10, *p* = 0.62) from dogs who were returned more than once. Intake age was associated with multiple return status (*X*_*2*_ = 14.94, *p* = 0.002) with significantly fewer puppies and more young adult dogs returned more than once. Breed groups also differed between one-off returns and multiple returns (*X*_*2*_ = 18.11, *p* = 0.01) with multiple-returns comprising more pit bull-type breeds and fewer sporting breeds. For cats, there were no significant differences between animals that were returned once and multiple returns. Overall, there was poor agreement between the first and second returning owners in terms of the reason provided for return (κ = 0.08, 95% CI 0.03–0.13, p = 0.002). The strongest agreement was seen between owners who returned animals due to the animal’s health (κ = 0.29, 95% CI 0.18–0.40, p < 0.001), although the strength of agreement was only considered ‘fair’.

### Associations between animal characteristics and likelihood of return

The likelihood of return was associated with intake age and breed group for dogs, and intake age for cats (Table 2). Adult dogs (> 2–8 years) had the highest likelihood of return, with an odds ratio of 3.40 (95% CI 2.88–4.01), followed by young adults and senior dogs who had an odds ratio of 2.90 (95% CI 2.47–3.41) and 2.24 (95% CI 1.64–3.06), respectively. For cats, senior cats had the greatest likelihood of return compared with kittens (OR 4.97, 95% CI 3.34–7.40), followed by adult cats (OR 4.10, 95% CI 3.27–5.13) and young adult cats (OR 3.02, 95% CI 2.39–3.80) and. Considering breed, dogs in the toy breed group were 65% less likely to be returned following adoption compared with herding breeds (OR 0.35, 95% 0.26–0.47) and terriers were 35% less likely to be returned compared with herding breeds (OR 0.65, 95% CI 0.49–0.86). Intake type was not associated with the likelihood of return for dogs or cats.

**Table 2:** Logistic regression models describing the risk of return and euthanasia based on animal characteristics.

Characteristics	Risk of return		Return reason^a^		Outcome^b^	
	Odds ratio (95% CI)	P value	Odds ratio (95% CI)	P value	Odds ratio (95% CI)	P value
***Dogs***						
**Initial intake age**						
Puppy (≤ 6 months)	Reference	–	Reference	–	Reference	–
Young adult (> 6 months–2 years)	2.90 (2.47–3.41)	< 0.001*	1.70 (1.25–2.32)	0.001*	2.35 (1.21–4.56)	0.01*
Adult (> 2–8 years)	3.40 (2.88–4.01)	< 0.001*	1.85 (1.35–2.54)	< 0.001*	3.94 (2.04–7.59)	< 0.001*
Senior (8 + years)	2.24 (1.64–3.06)	< 0.001*	0.79 (0.44–1.42)	0.43	3.73 (1.36–10.22)	0.01*
**Sex**^**c**^	1.09 (0.98–1.22)	0.11	1.03 (0.84–1.26)	0.81	1.40 (1.04–1.90)	0.03*
**Return frequency**^**d**^	–	–	–	–	1.65 (1.15–2.37)	0.01*
**Return reason**^**a**^	–	–	–	–	4.14 (2.83–6.05)	< 0.001*
**Intake type**						
Surrender	Reference	–	Reference	–	Reference	–
Stray	0.99 (0.83–1.21)	0.99	0.80 (0.55–1.14)	0.22	0.61 (0.36–1.01)	0.05
Seized	1.06 (0.88–1.27)	0.53	0.88 (0.62–1.25)	0.49	0.76 (0.47–1.21)	0.25
Transfer from another animal shelter	1.03 (0.79–1.34)	0.84	0.88 (0.53–1.44)	0.60	1.05 (0.54–2.01)	0.90
**Breed group**						
Herding	Reference	–	Reference	–	Reference	–
Hound	1.15 (0.93–1.43)	0.19	0.59 (0.39–0.88)	0.01*	1.05 (0.55–2.00)	0.88
Non-sporting	0.90 (0.59–1.37)	0.62	0.86 (0.38–1.94)	0.79	0.93 (0.24–3.53)	0.91
Pit bull-type	1.09 (0.89–1.33)	0.41	0.72 (0.49–1.06)	0.10	2.60 (1.47–4.61)	0.001*
Sporting	0.85 (0.68–1.06)	0.14	0.62 (0.41–0.95)	0.02*	1.02 (0.51–2.03)	0.95
Terrier	0.65 (0.49–0.86)	0.002*	0.84 (0.49–1.46)	0.53	1.23 (0.53–2.83)	0.63
Toy	0.35 (0.26–0.47)	< 0.001*	0.92 (0.50–1.66)	0.72	0.67 (0.25–1.84)	0.44
Working	0.99 (0.74–1.33)	0.94	0.97 (0.55–1.71)	0.90	1.86 (0.85–4.08)	0.12
***Cats***						
**Initial intake age**						
Kitten (≤ 6 months)	Reference	–	Reference	–		
Young adult (> 6 months–2 years)	3.02 (2.39–3.80)	< 0.001*	1.52 (0.96–2.38)	0.07		
Adult (> 2–8 years)	4.10 (3.27–5.13)	< 0.001*	2.17 (1.38–3.41)	0.001*		
Senior (8 + years)	4.97 (3.34–7.40)	< 0.001*	2.93 (1.30–6.58)	0.01*		
**Sex**^**b**^	1.10 (0.93–1.31)	0.27	1.02 (0.73–1.44)	0.90		
**Intake type**						
Surrender	Reference	–	Reference	–		
Stray	1.09 (0.82–1.44)	0.55	1.35 (0.78–2.35)	0.29		
Seized	1.24 (0.93–1.67)	0.15	1.65 (0.92–2.94)	0.09		
Transfer from another animal shelter	0.71 (0.46–1.08)	0.11	0.77 (0.34–1.75)	0.53		

*Denotes statistical significance (*p* < 0.05).

^a^Owner-based return reasons were coded as the reference category.

^b^Reflects the most recent outcome for animals that were returned multiple times.

^c^Females were coded as the reference category.

^d^Return frequency was coded as dichotomous based on animals who were returned once and animals who were returned more than once. Single returns were coded as the reference category.

### Return reasons

**Figure 1:**
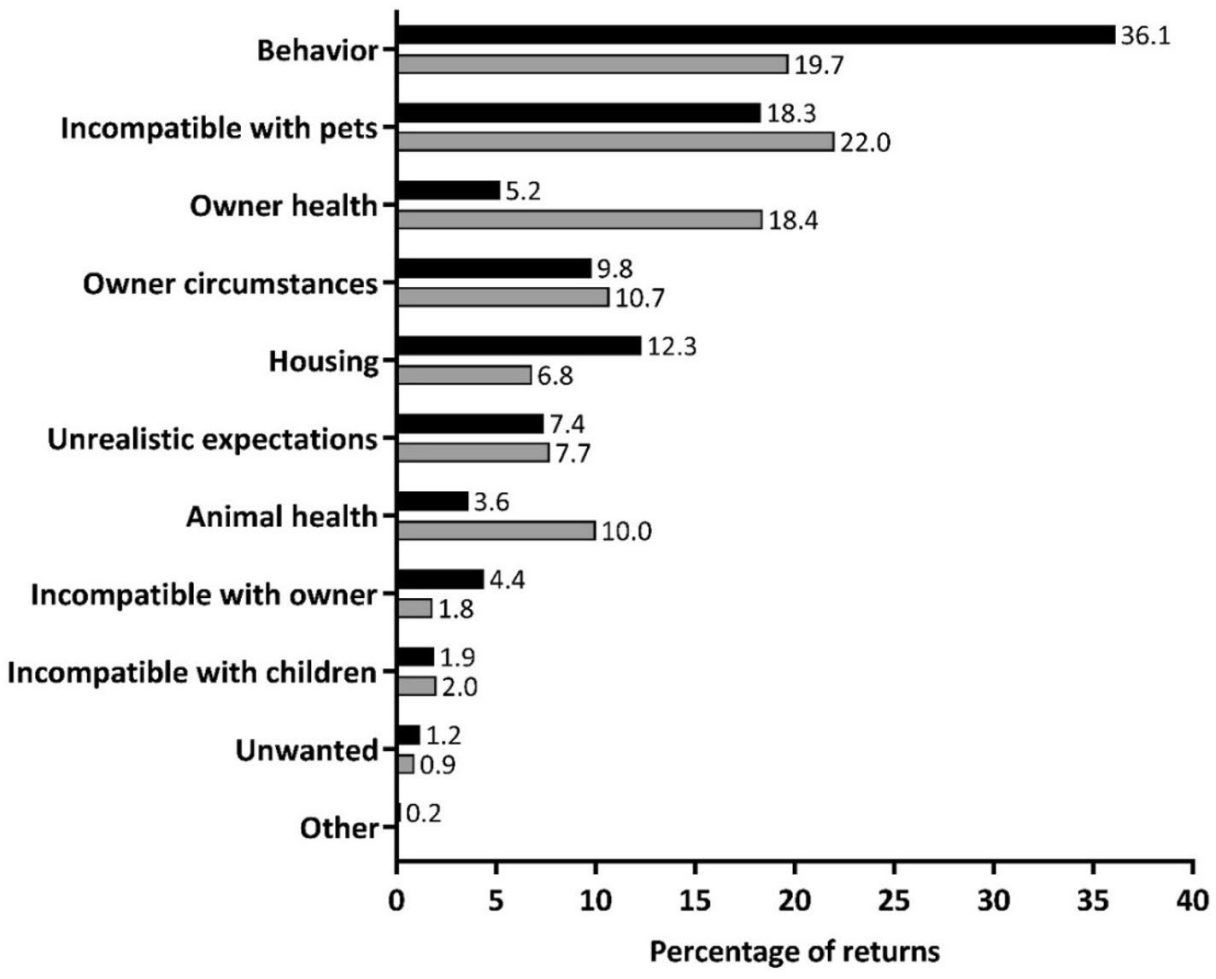
Categorized reasons for returns, including the first return for animals that were returned multiple times. Black bars represent dogs (*n* = 1627) and grey bars represent cats (*n* = 559).

The return reasons for dogs and cats are presented in Fig. 1 and Table 3. Behavioral issues (36.1%) were the most common reason for return for dogs, followed by incompatibility with existing pets (18.3%). For cats, the most common return reason was incompatibility with existing pets (22.0%), followed by behavioral issues (19.7%) and owner’s health, including allergies (18.4%). Rabbits were mainly returned due to behavioral issues (*n* = 4, 26.7%), incompatibility with existing pets (*n* = 3, 20.0%) and housing issues (*n* = 3, 20.0%). All guinea pigs were returned due to incompatibility with pets (*n* = 4). The two pigs were returned due to behavior and incompatibility with pets, the mice were returned due to being unwanted (*n* = 2) and the hamster was returned due to unrealistic expectations.

**Table 3:** Reasons for returned adoptions including first return only for multi-return animals (*n* = 2186).

Return reason	Dog (*n* = 1627)		Cat (*n* = 559)	
	*n*	%	*n*	%
**Behavior**^**a**^	587	36.08	110	19.69
Aggression to animals	73	4.49	8	1.43
Aggression to people	33	2.03	6	1.07
Behavior issues^b^	218	13.40	44	7.87
Bite history	26	1.60	2	0.36
Chases animals	6	0.37	0	0.00
Destructive	59	3.62	3	0.54
Escapes	23	1.41	2	0.36
Needs too much attention	24	1.48	7	1.25
Not friendly	0	0.00	3	0.54
Not housebroken/house soiling	10	0.61	12	2.15
Temperament	9	0.55	7	1.25
Too active	93	5.72	12	2.15
Too noisy	4	0.25	4	0.72
Unable to train	9	0.55	0	0.00
**Owner circumstances**^c^	160	9.84	60	10.73
Cannot afford	8	0.49	6	1.07
Change in lifestyle	19	1.17	7	1.25
Divorce/separation	2	0.12	1	0.18
New baby	5	0.31	0	0.00
Not enough time	68	4.18	16	2.86
Personal problems	53	3.26	25	4.47
Too many animals	0	0.00	2	0.36
Travel	5	0.31	3	0.54
**Health of owner**^c^	84	5.16	103	18.42
Allergic to animal	46	2.83	87	15.56
Health of owner/family	38	2.33	16	2.86
**Health of animal**^**a**^	59	3.62	56	10.02
**Housing**^**c**^	200	12.28	38	6.80
Inadequate housing/yard	29	1.78	3	0.54
Landlord issues	128	7.86	16	2.86
No home	7	0.43	2	0.36
Moving	36	2.21	17	3.04
**Not compatible with children**^**a**^	31	1.90	11	1.97
Doesn't like children	29	1.78	11	1.97
Plays rough with children	2	0.12	0	0.00
**Not compatible with pets**^**a**^	298	18.31	123	22.01
Doesn't like other pets	160	9.83	64	11.45
Pets in home didn't like	138	8.48	59	10.56
**Not compatible with owner**	71	4.35	10	1.79
Litterbox odor^c^	0	0.00	1	0.18
Sheds^a^	1	0.06	0	0.00
Too big^a^	15	0.92	2	0.36
Too much responsibility^c^	48	2.95	7	1.25
Wrong sex^c^	1	0.06	0	0.00
**Other**^**c**^	3	0.18	0	0.00
Abandoned by owner	1	0.06	0	0.00
Court order	1	0.06	0	0.00
Insurance restrictions	1	0.06	0	0.00
**Unrealistic expectations**^**c**^	121	7.44	43	7.69
**Unwanted**^**c**^	19	1.17	5	0.89

^a^Animal-based return reason.

^b^Includes multiple behavioral issues.

^c^Owner-based return reason.

**Figure 2:**
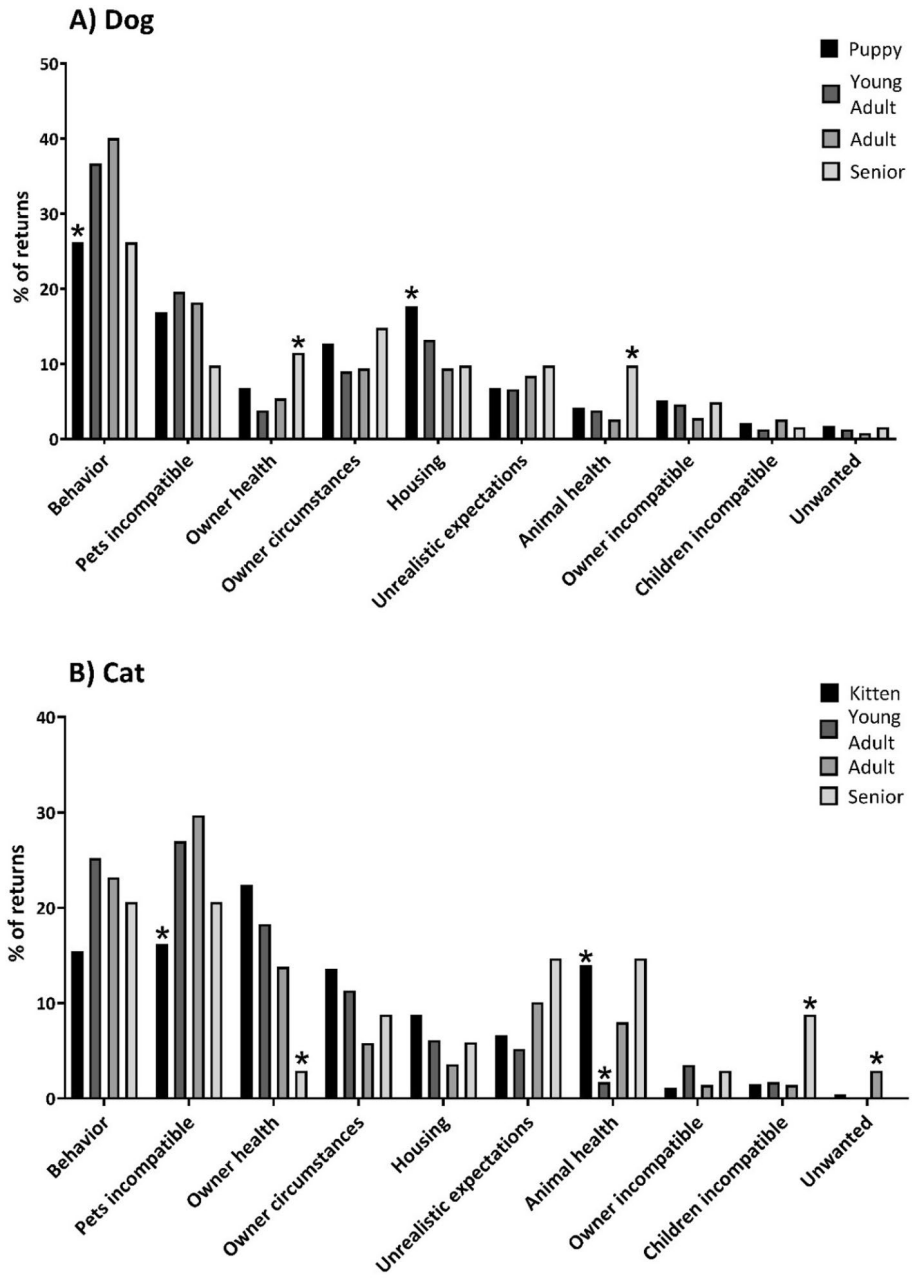
Categorized return reasons by age group. *Denotes statistical significance (*p* < 0.05).

Return reasons differed across age groups for both dogs (*X*_*2*_ = 57.96, p = 0.002) and cats (*X*_*2*_ = 69.91, p < 0.001) as shown in Fig. 2. Breed groups were associated with return reasons for dogs (*X*_*2*_ = 99.72, *p* = 0.01). Hounds were returned more frequently than other breed groups due to the owner’s health or owner circumstances, while toy breeds were returned more frequently for the animal’s health and incompatibility with children. Sporting breeds were more often ‘unwanted’. Sex was not significantly associated with return reasons for dogs (*X*_*2*_ = 4.58, *p* = 0.92) or cats (*X*_*2*_ = 11.32, *p* = 0.26).

**Figure 3:**
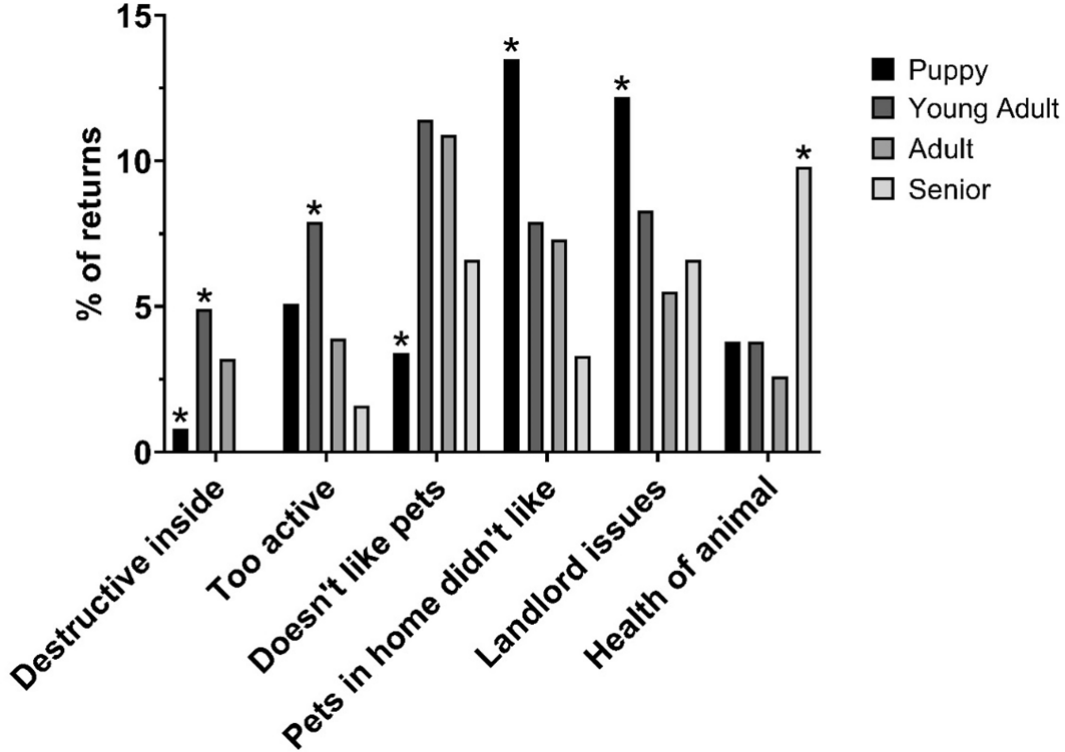
Differences in non-categorized return reasons for dogs based on age group. *Denotes statistical significance (*p* < 0.05).

Canine return reasons were analyzed individually if more than 50 owners provided the return reason, which showed age at intake was significantly associated with the following reasons: destructive inside, too active, doesn’t like pets, pets in home didn’t like, landlord issues and health of animal (Table 4). The differences in return reasons based on age are shown in Fig. 3. Breed group was associated with ‘aggression to animals’ in dogs with significantly more working breeds and fewer terrier breeds returned for aggression to animals.

**Table 4:** Individual return reasons (*n* > 50) by sex, age, and breed among dogs.

Return reason	Sex		Age at intake		Breed group	
	*X*_*2*_	P	*X*_*2*_	P	*X*_*2*_	P
Aggression to animals	1.55	0.23	7.70	0.05	19.85^a^	0.01*^b^
Behavior issues	1.05	0.31	7.39	0.06	3.83	0.80
Destructive inside	0.03	0.89	11.47	0.01*	7.77^a^	0.35
Doesn’t like pets	0.67	0.45	14.50	0.003*	12.71	0.08
Pets in home didn’t like	0.27	0.60	10.99	0.01*	12.51	0.09
Too active	2.20	0.16	11.94	0.01*	8.01	0.33
Health of animal	0.09	0.79	8.82	0.03*	9.55^a^	0.18
Landlord issues	2.03	0.15	10.79	0.01*	9.45	0.22
Not enough time	0.14	0.71	2.55	0.47	12.47^a^	0.07^b^
Personal problems	2.33	0.13	3.26	0.34	1.86^a^	0.97
Unrealistic expectations	0.03	0.93	2.29	0.51	5.72	0.57

*Denotes statistical significance (*p* < 0.05).

^a^Fisher–Freeman–Halton Exact Test due to > 20% of cells with an expected count below 5.

^b^Monte Carlo approximation method used based on 10,000 sampled tables.

Logistic regression models including return reason as a dichotomous variable (owner or animal-based) mirrored the results of the Chi-Square analysis, highlighting a significant association between return reason and age at intake for both dogs and cats, and breed for dogs only (Table 2).

### Outcome following return

Most returned animals were re-adopted (Table 1), including 80.3% of dogs and 90.2% of cats. Outcome was associated with intake age, breed group, sex, return reason and return frequency for dogs (Table 2). Return reason was the strongest predictor of euthanasia followed by age.

## Discussion

To our knowledge, this study is the largest to date to investigate post-adoption returns in a sample of 23,932 adopted animals with a 9.2% return rate. Dogs were the most frequently returned species with a return rate of 16.3%. Cats had a lower return rate of 4.2% and almost one in ten rabbits were returned post-adoption. Of those returned, most animals were returned once during the study period although 14.3% of returned animals were returned more than once. Age at intake and breed (dogs only) predicted the odds of return, return reasons and post-return outcome.

The likelihood of return increased significantly for both dogs and cats over the age of 6 months. Adult dogs were over three times more likely to be returned, followed by young adults and senior dogs who had 2.9 and 2.2 times the odds of being returned compared with puppies. The elevated risk of return in adult dogs may be explained by differences in behavior based on age of acquisition. Research has shown that as the age at acquisition increases, so too does the risk of resource guarding^23^, and destructive behaviors^24^. However, in both studies other variables had a greater influence on the likelihood of behavioral problems, such as the dog’s sex^24^ and owner’s behavior^23^. Variations in early life experience may also produce behavioral differences and therefore, influence the likelihood of return^25^. The lower odds of return among senior dogs may be attributed to reduced exercise and training requirements, particularly if the dog has lived in a home previously. This finding is somewhat at odds with previous reports that older animals may be at greater risk of relinquishment due to ill-health^26^ which speaks to the possible differences between the experience of a returned adoption and an owner relinquishment. Interestingly, young adult dogs comprised a higher proportion of multiple-returns than the other age groups, perhaps reflecting an adolescent phase characterized by increased conflict behavior^27^. Qualitative research in the field has documented a similar trend. Shore^17^ found some returning owners indicated they would adopt a dog of a different age in the future, although the direction of change was split. Several respondents were interested in acquiring an older dog, while others would acquire a puppy. One respondent said she would adopt either a very young dog or an older dog in the future but would avoid dogs in between^17^. For cats, the risk of return increased considerably for each age group. Young adult cats, adult cats and senior cats were 3.0, 4.1 and 5.0 times more likely to be returned than kittens, respectively. The willingness of cats to interact with humans has been shown to decrease with age which may hinder the development of the human-cat bond and increase the risk of return^28^. Kittens are also more adaptable to novel environments^29^.

Breed group influenced the likelihood of return for dogs in that toy and terrier breeds were significantly less likely to be returned. The association between breed group and risk of return may be attributable to the dog’s size as previous research has shown medium and large dogs are more likely to be returned than small dogs^8^. We could not test this hypothesis due to a lack of data regarding dogs’ weight. Breed group was also associated with the frequency of return with pit bull-type breeds comprising a higher proportion of multiple-returns than other breed groups.

Behavioral issues were a key reason for return of both dogs and cats, which parallels the current body of evidence and affirms the importance of animal behavior in the development of a positive human-animal relationship^8, 9, 12, 18^. Charleston Animal Society provides a multitude of behavioral support services for adopters, although data regarding the utilization of these services was not available. Recent reports indicate relatively few owners accept behavioral support^18, 30^. At a shelter in Texas, less than half of returning owners utilized behavior support services, with 18% of dog adopters and 7% of cat adopters contacting the behavior team more than once prior to returning their pet^18^. Future studies on the use and efficacy of postadoption behavioral support programs in reducing returns would be of great value to the field. Incompatibility with existing pets contributed to approximately 20% of cat and dog returns; a higher rate than previous reports which ranged between 3^17^ and 19%^5^. Our findings emphasize the importance of considering existing pets during adoption counselling. Cat adopters with existing pets may also benefit from adopting a kitten rather than an older cat as kittens had a significantly lower rate of return due to incompatibility with existing pets. The ideal age group for dog adopters with existing pets is more complex as puppies were returned at a lower rate for ‘doesn’t like pets’ but a higher rate for ‘pet in the home didn’t like’. Allergies were also a significant driver of returns for cats, contributing 15% of all cat returns which is broadly comparable to previous studies^5, 17, 19^.

Age at intake was associated with return reasons for both dogs and cats. Considering owner- and animal-related return reasons as a dichotomous variable, we found young adult and adult dogs were more likely to be returned for animal-related reasons than puppies. Specifically, puppies were returned at a lower rate for behavior issues, namely ‘destructive inside’, while young adult dogs were returned more frequently for ‘destructive inside’ and ‘too active’. Again, the increased rate of behavioral returns among young adults could be attributed to a phase of adolescent behavior^27^. Adopters’ expectations for ownership may also vary between age groups. Most prospective dog owners expect some difficulties with dog training and behavior^31^, although puppy adopters may anticipate more ‘puppy-like’ behavior, such as destructive tendencies^32^. On the contrary, puppies were returned at a higher rate for housing issues, namely ‘landlord issues’. Perhaps, puppy adopters encountered more house-training issues that led to post-adoption returns. It is also possible that puppy adopters were more likely to impulsively adopt without approval from their landlord, but further research is needed to confirm this hypothesis. We also found senior dogs were returned at a higher rate due to the health of the owner or animal. Older adults may be more inclined to adopt senior dogs due to their perceived lower training and exercise needs, although further research is needed to support this hypothesis. Among cats, we found adult and senior cats had an increased likelihood of return due to animal-related reasons. As described above, this may be related to the reduction in sociality with increasing age^28^. Further analyses of the categorized return reasons showed a higher proportion of kitten returns occurred due to health concerns, possibly upper respiratory infections (URI). URIs are common in the shelter environment and affect kittens at a higher rate due to their increased susceptibility^33, 34^. Senior cats were returned at a higher rate for incompatibility with children which is conceivable as older cats are reported to have the least satisfactory child-cat relationships^35^.

Return reasons also differed based on breed group in dogs. Sporting breeds and hounds were less likely to be returned for animal-based reasons than herding breeds, with hounds returned at a higher rate due to the owner’s health and circumstances, and sporting breeds returned at a higher rate for being unwanted. The categorization of return reasons also showed toy breeds were returned more frequently due to the animal’s health and incompatibility with children. Considering the individual return reasons, working breed dogs were returned at a higher rate for aggression to animals which is unsurprising given one of the historical roles of the breed group is guarding^36^. Future, prospective research focused on the influence of dog breed on return reasons would enhance our understanding of these associations.

Most returned animals were re-adopted, including 80% of dogs and 90% of cats. The likelihood of re-adoption was associated with intake age, return reason, return frequency and sex for dogs. Age was a strong predictor of euthanasia with adult and senior dogs having approximately four times greater odds of euthanasia, and young adult dogs displaying two times greater odds of euthanasia compared with puppies. Return reason was also a significant predictor of euthanasia for dogs. Dogs who were returned for animal-related reasons were more than four times more likely to be euthanized than dogs returned for owner-related factors. Older animals and those returned for animal-related reasons may have had behavioral and/or medical concerns that meant they were unsuitable for rehoming. Animal behavior and health are also important considerations for adopters when choosing an animal^37, 38^ and animals relinquished for behavioral problems, old age, illness, and injury are less likely to be adopted^39^. Dog breed was also associated with outcome. Pit bull-type breeds were more than two and half times more likely to be euthanized post-return than other breed groups, replicating the findings of previous research^39–41^. The process of breed designation is undoubtedly subject to potential error. Shelter staff are inconsistent in their breed designations, and significant differences have been found between shelter breed labels and DNA analysis^42–44^. Irrespective of the accuracy of designated breeds, breed labels have been identified as an important attribute in adoptability^40, 45^. Dogs labelled as pit bulls have been shown to spend longer in the shelter compared with phenotypically similar dogs labelled as alternative breeds. In the same study, participants rated pit bulls as less intelligent, less approachable, less friendly, less adoptable, and more aggressive than Border Collies and Labrador Retrievers^40^. Pit bull-type breeds may also differ behaviorally from other breeds, although current data is mixed. Some research suggests pit bulls show higher levels of interdog aggression^46^, hyperactivity, impulsivity and compulsive behavior^47^ while other studies have found the behavior of pit bulls was no worse than other breeds^48, 49^. Although it is unclear whether the association between pit bull-type breeds and euthanasia post-return is attributable to the breed-specific characteristics of the dog or the perceptions of the dog based on breed label, future adoption counselling and post-adoption support services could target this group of at-risk dogs to potentially reduce returns and post-return euthanasia rates. Shelters may also see differences in post-return outcomes with removal of the breed label. Male dogs and dogs who were returned more than once also had slightly elevated odds of euthanasia.

One limitation of this study is the reliance on owner reports of return reasons. Owners may provide unreliable information at the time of return due to social desirability bias, in which individuals give answers that they believe will be viewed more favorably by others, or to reduce the animal’s risk of euthanasia. Research on the topic has produced mixed results. Some data indicates that relinquishing owners reliably report their dog’s behavior at the time of relinquishment^50^ while other studies have found inconsistencies between relinquishing owner reports and dog behavior^51, 52^. Owner-reported return reasons are likely to also be influenced by the adopter’s expectations for ownership, ownership behaviors, perception of animal behavior and tolerance of behavioral problems^8, 21^. For example, animal behavior that is construed as normal by one adopter could be considered problematic by another^53^. This phenomenon has been documented previously whereby some owners do not report behaviors as a problem despite reporting occurrences of the behavior itself. Research has found dog owners who are employed/students, use positive reinforcement training methods only, do not attend puppy classes or own small dogs are less likely to report problem behaviors as a problem^54^. The multifactorial nature of returns means the categorization of return reasons is also subject to potential error. For instance, a dog that is returned for being ‘too active’ may have arousal and hyperactivity issues that make it difficult for the owner to manage safely, or the dog’s activity level may simply be incompatible with the owner’s lifestyle. Due to the retrospective nature of the study, we were unable to decipher such ambiguities. Future, prospective research should aim to disentangle the owner- and animal-related factors that contribute to post-adoption returns to increase our understanding of return reasons. The retrospective study design also prevented the inclusion of additional variables, such as animal behavior, size, ownership behaviors, and owner-animal attachment, that likely influence the human–dog relationship and the risk of return^49^. In particular, dog size may affect the likelihood of return^8^ and could be confounding the results surrounding breed. Charleston Animal Society operates an open adoption policy and encourages adopters to return animals directly to the shelter if necessary, although it is possible that we misclassified animals who were rehomed through other avenues. The uncertain history of many animals entering the shelter meant some variables were subject to potential error. To reduce the risk of misclassification, we used categorical groupings where appropriate. Finally, despite the large sample size of the study, the generalizability of the findings is limited as the data were collected from a single facility that likely differs from other shelters. Charleston Animal Society provides post-adoption support services which may have reduced return rates by helping new owners to manage behavioral or medical difficulties. On the other hand, the open return policy could have increased return rates as owners were aware that they could return the animal without any ramifications.

Through this study, we have identified groups of the shelter population that experience greater risk of post-adoption return. These data are crucial to promote successful adoptions and improve animals’ outcomes as they highlight opportunities for targeted interventions and aid in the early recognition of adopter-animal mismatches. Our results also pave the way for future research focused on the usefulness of interventions, such as behavioral support services, in reducing returns.

## Conclusion

The most common reasons for returned adoptions at this large animal shelter in South Carolina were behavioral issues and incompatibility with existing pets. Taken together, our findings indicate that adult (> 2–8 years) and young adult dogs (6 months-2 years) were most likely to be returned following adoption, primarily due to animal-based reasons, such as behavior. Adult dogs had the greatest odds of euthanasia post-return at almost four times that of puppies. Toy and terrier dogs were less likely to be returned, while pit bull-type breeds were more likely to be returned multiple times and more likely to be euthanized post-return. Our study adds to a growing body of evidence that highlights the importance of animal behavior in the retention of newly adopted animals and the development of the human-animal relationship. Our findings also provide useful direction for future adoption counselling and allocation of resources, including post-adoption support services.

## Methods

### Animal shelter characteristics

Charleston Animal Society is a large, open admission shelter located in South Carolina, USA. The shelter is the only open access shelter in the region and took in approximately 3500 dogs and 4700 cats per year between 2015 and 2019. Most animals that entered the shelter were strays, including 72% of dog and 89% of cat intakes. The remainder of shelter intakes were mostly owner relinquishments, including 24% of dog and 10% of cat intakes. Charleston Animal Society operates an open adoption policy meaning adopters can adopt and return animals to the shelter without judgement or ramifications. If the animal is returned within 30 days of adoption, adopters receive a refund in the form of a voucher for future adoptions (except for animals adopted during fee-waived promotions). The shelter also provides post-adoption behavioral and veterinary support services. All adopted animals are eligible for a free veterinary check-up at local veterinary clinics and adopters can seek behavioral advice from the shelter’s behavior team if needed. In most cases, adopters of animals with identified behavior problems also receive post-adoption follow-up phone calls from the behavior team.

### Variables

For all animals adopted between 1st January 2015 and 31st December 2019, sex, known or estimated date of birth, breed (dogs only), intake date, intake type and adoption date were extracted from the electronic shelter records (PetPoint Data Management System, Version 5, Pethealth Software Solutions Inc., USA). Charleston Animal Society consented to the sharing of data as the data are not publicly available. Animals returned to the shelter within 30 days of adoption were classified as a ‘return’ in the shelter’s electronic database. To capture animals returned outside of the 30-day window, we extracted data for animals relinquished to the shelter within six months of the adoption where the person’s ID was the same between the adopter and the relinquishing owner. We then extracted return/relinquishment reason, return/relinquishment date, return/relinquishment outcome date, and return/relinquishment outcome (adoption, euthanasia, return to owner/guardian, transferred to another animal shelter, died, return to field).

Length of stay was calculated as the number of days between the animal’s intake date and outcome date, including any time spent in a foster home. Age at intake was calculated as the number of months between the animal’s recorded date of birth and first intake date. For returned animals, age at intake was calculated as the number of months between date of birth and date of initial intake (prior to the first return between 2015 and 2019). Post-return intake age was calculated as the number of months between the animal’s date of birth and return date. Intake age was then categorized as puppy/kitten (< 6 months), young adult (> 6 months–2 years), adult (> 2–8 years) and senior (> 8 years). Primary breed was determined at intake based on staff opinion due to the dog’s phenotypic characteristics or the breed provided by the relinquishing owner. Staff could include a secondary breed in the electronic record or select ‘mix’ to indicate the dog was a mixed breed. Dogs were then categorized based on their primary breed designation in accordance with the American Kennel Club’s breed groups as herding, hound, non-sporting, sporting, terrier, toy and working^55^, with one additional category for pit bull-type breeds^40^. Dogs listed as Catahoula Leopard dog and Treeing Tennessee Brindle were coded as hounds^56^. The full list of breeds and breed groups are provided in Supplementary Table S1. Spay/neuter status was not included in the analyses as the animal shelter mandates that all animals are sterilized prior to adoption.

### Statistical analyses

Descriptive statistics were calculated for intake age group, breed group (dogs only), intake type, length of stay and outcome type. Length of stay was assessed for normality using the Shapiro–Wilk test and visual inspection of histograms. Although the data were not normally distributed, independent t-tests were used to compare length of stay between returned and non-returned animals as they are robust for skewed data sets with large sample sizes^57^. Paired t-tests were used to compare length of stay at initial intake and post-return intake for returned dogs and cats. We then used Pearson’s Chi-Square tests to compare intake age group, intake type, sex, and breed group (dogs only) between returned and non-returned animals. Animals with return listed as their first intake type between 2015 and 2019 were excluded from analyses for intake type (*n* = 14 dogs, *n* = 1 cats). Pearson’s Chi-Square tests were used to compare return reasons, return frequency and outcome type by intake age group, sex, and breed groups (dogs only). If > 20% of cells had expected values below 5, Fisher-Freeman-Halton Exact test were used. Return reasons with *n* ≥ 50 were also considered individually for dogs only. Post-hoc analyses were conducted using standardized residuals^58^. For animals returned more than once, Fleiss’ kappa was used to test the strength of agreement between the return reasons provided by the first and second returning owners. Binary logistic regression models were used to describe the likelihood of return (returned/not returned), return reason (owner-based/animal-based) and outcome (adopted/euthanized) based on intake age group, sex, breed group (dogs only) and return frequency (outcome only). Outcome was investigated among dogs only due to the small number of cats that were euthanized (*n* = 18). Statistical analyses were performed in IBM SPSS Statistics for Windows, version 24. Statistical significance was set at *p* < 0.05 without adjustment for multiple comparisons^59, 60^.

## Supplementary Information

Supplementary Information

## Data Availability

The data governance arrangements for the study do not allow us to redistribute Charleston Animal Society data to other parties.
