# Supplementary material for: Characterizing unsuccessful animal adoptions: age and breed predict the likelihood of return, reasons for return and post-return outcomes
Source: Sci Rep. 2021 Apr 13;11:8018. doi: 10.1038/s41598-021-87649-2 (PMC8044234; doi:10.1038/s41598-021-87649-2)
Supplement: Supplementary file 1 — Supplementary Information [file 41598_2021_87649_MOESM1_ESM.pdf]

Characterizing unsuccessful animal adoptions: Age and breed predict the likelihood of return, reasons for return and post-return outcomes

Lauren Powell [a], Chelsea Reinhard [a], Donya Satriale [b], Margaret Morris [b], James Serpell [a†], Brittany Watson[a†]

[†]Joint senior author

Supplementary Table 1. Breeds and breed groups of all dogs adopted between 2015 and 2019 (*n*=9996)

Breed group	*n*	Breed assigned by shelter
Herding	982	American Blue Heeler, Australian Cattle Dog, Australian Kelpie, Australian Shepherd, Australian Shepherd (Miniature), Belgian Malinois, Belgian Tervuren, Border Collie, Canaan, Rough Collie, Smooth Collie, Dutch Shepherd, English Shepherd, German Shepherd, German Shepherd (King), McNab Herding Dog, Shepherd, Swedish Vallhund, Welsh Corgi (Cardigan), Welsh Corgi (Pembroke)
Hound	1660	Basenji, Basset Hound, Beagle, Bloodhound, Catahoula Leopard dog, Coonhound, Coonhound (American English), Coonhound (Black and Tan), Coonhound (Bluetick), Coonhound (Redbone), Coonhound (Treeing Walker), Dachshund (Miniature Long Haired), Dachshund (Miniature Smooth Haired), Dachshund (Miniature Wire Haired), Dachshund (Standard Long Haired), Dachshund (Standard Smooth Haired), Dachshund (Standard Wire Haired), American Foxhound, English Foxhound, Greyhound, Harrier, Hound, Irish Wolfhound, Norwegian Elkhound, Plott Hound, Rhodesian Ridgeback, Schweizer Laufhund (Swiss Hound), Thai Ridgeback, Whippet, Treeing Tennessee Brindle
Non-sporting	173	American Eskimo, Bichon Frise, English Bulldog, French Bulldog, Victorian Bulldog, Chinese Shar-Pei, Chow Chow, Dalmatian, Finnish Spitz, Lhasa Apso, Mexican Hairless, Poodle (Standard), Shiba Inu, Tibetan Spaniel, Boston Terrier
Sporting	1825	Brittany, Wirehaired Pointing Griffon, Pointer, German Shorthaired Pointer, German Wirehaired Pointer, Retriever, Chesapeake Bay Retriever, Flat-Coated Retriever, Golden Retriever, Labrador Retriever, Nova Scotia Duck Tolling Retriever, English Setter, Spaniel, American Cocker Spaniel, Boykin Spaniel, English Cocker Spaniel, English Springer Spaniel, Smooth Haired Vizsla, Wire Haired Vizsla, Weimaraner
Terrier	761	Feist, Schnauzer (Miniature), Terrier, Airedale Terrier, Cairn Terrier, Fox Terrier (Smooth), Irish Terrier, Jack Russell Terrier, Patterdale Terrier, Rat Terrier, Scottish Terrier, Silky Terrier, Soft Coated Wheaten Terrier, Welsh Terrier, West Highland White Terrier, Yorkshire Terrier

Toy	970	Chihuahua (Long Coat), Chihuahua (Short Coat), Brussels Griffon, Havanese, Italian Greyhound, Japanese Chin, Maltese, Miniature Pinscher, Papillon, Pekingese, Pomeranian, Poodle (Miniature), Poodle (Toy), Pug, Shih Tzu, Cavalier King Charles Spaniel, Manchester Terrier
Working	483	Akita, Alaskan Husky, Alaskan Malamute, Anatolian Shepherd, Bernese Mountain Dog, Boerboel, Boxer, Bullmastiff, Cane Corso, Doberman Pinscher, Dogue de Bordeaux, English Mastiff, Great Dane, Great Pyrenees, Greater Swiss Mountain Dog, Hovawart, Mastiff, Rottweiler, Saint Bernard, Samoyed, Schnauzer (Giant), Schnauzer (Standard), Siberian Husky
Pit bull-type	3037	Alapaha Blue Blood Bulldog, American Bulldog, Olde English Bulldogge, American Pit Bull Terrier, American Staffordshire Terrier, Bull Terrier, English Staffordshire Terrier, Pit Bull Terrier, Staffordshire Bull Terrier
Excluded	104	Bulldog (*n*=44), Black-Mouth Cur (*n*=7), Carolina Dog (*n*=37), Mixed Breed, Large (over 44 lbs fully grown) (*n*=1), Mountain Cur (*n*=16)
